# The association between glucose-related variables and plaque morphology in patients with ST-segment elevated myocardial infarction

**DOI:** 10.1186/s12933-020-01074-9

**Published:** 2020-07-08

**Authors:** Jinxin Liu, Shanjie Wang, Can Cui, Hengxuan Cai, Rong Sun, Weili Pan, Shaohong Fang, Bo Yu

**Affiliations:** 1 Department of Cardiology, The Second Affiliated Hospital of Harbin Medical University, Harbin, 150086 China; 2 The Key Laboratory of Myocardial Ischemia, Harbin Medical University, Ministry Education, Harbin, 150086 Heilongjiang China; 3 Department of Endocrinology and Metabolism, The Second Affiliated Hospital of Harbin Medical University, Harbin, 150086 China

**Keywords:** Admission glucose, Plaque erosion, Plaque rupture, ST-segment elevated myocardial infarction

## Abstract

**Background:**

Plaque rupture (PR) and plaque erosion (PE) are main causes of acute myocardial infarction with different demographic and histology characteristics and need different treatment strategy. PR and PE can be identified with optical coherence tomography (OCT) accurately, but convenient and effective noninvasive markers for them are rarely found. History of diabetes mellitus (DM) was reported to be a potential predictor of PR in ST-segment elevated myocardial infarction (STEMI) patients, but the predictive value of other glucose-related variables for it is still uncertain. Present study aimed to clear the relationship between some glucose-related variables and plaque morphology in patients with STEMI.

**Methods:**

We consecutively enrolled 872 STEMI patients and divided them into PR group (n = 616) and PE group (n = 256) based on OCT diagnostic criteria. The relationship of glucose-related variables, including random plasma glucose on admission (ARPG), glycosylated hemoglobin (HbA1c), post-PCI fasting plasma glucose (PFPG), DM history, glucose variable tendency (GVT) and the acute-to-chronic glycemic ratio (A/C), to the PR risk of STEMI patients was analyzed. The correlation between the glucose-related variables and plaque morphology was analyzed meanwhile.

**Results:**

Among the glucose-related variables, ARPG and GVT were confirmed to be independent predictors for PR after adjusting for other traditional risk factors in nondiabetic patients. The higher the ARPG level, the more PR risk the STEMI patients had. And high HbA1c and APPG were demonstrated to have a weak and positive correlation with lipid constituents and stenosis degree of culprit vessel.

**Conclusions:**

Compared to HbA1c, DM history, and some other glucose-related variables, ARPG and GVT were risk factors for PR in STEMI patients, especially those without DM. And high HbA1c and ARPG were positively correlated with the development of vulnerable plaque in culprit vessels.

*Trial registration* Present study is a retrospective one and the population came from the EROSION study of our center previously. It was approved by the Ethics Committee of the Second Affiliated Hospital of Harbin Medical University (Approval reference number, KY2017-249), and all patients provided written informed consent prior to the inclusion in the study and the investigation conformed to the principles outlined in the Declaration of Helsinki.

## Background

Acute myocardial infarction (AMI) is the main cause of life threat in patients with coronary heart disease (CHD). Many studies have shown the positive correlation between the bad outcome of AMI and glucose-related variables, such as serum glycosylated hemoglobin (HbA1c) [1, 2], the acute-to-chronic glycemic ratio (A/C) [3], both admission hyperglycemia (AH) and post-admission hyperglycemia [4, 5]. Plaque erosion (PE) and plaque rupture (PR), the two main causes of AMI, present different lesion morphology distinctively [6]. Same as hyperglycemia and diabetes mellitus (DM), lesion morphology may affect clinical outcomes of patients with ST-elevated myocardial infarction (STEMI) [7]. And different treatment strategy should be made for STEMI patients with PR and PE [8]. PR and PE can be identified with optical coherence tomography (OCT) accurately [9], but convenient and effective noninvasive markers for them are rarely found. A study in our center showed that the history of DM is a potential predictor of lesion morphology [10]. However, the association between other glucose-related variables and the lesion morphology causing STEMI patients is still unclear. Thus, we analyzed the association between some glucose-related variables [included HbA1c, admission glucose (ARPG), post-PCI fasting plasma glucose (PFPG), DM history, A/C, and glucose variation tendency (GVT)] and plaque morphology by using optical coherence tomography (OCT) in patients with ST-elevating myocardial infarction caused by PR or PE.

## Methods

### Design and population

This study is a subanalysis of the EROSION study [11]. For studying the association between glucose-related variables and plaque model of STEMI patients, a total of 1005 patients hospitalized within 24 h of symptom onset in the intensive cardiology care unit of the second affiliate hospital of Harbin Medical University who underwent coronary angiography and optical coherence tomography for STEMI between August 2014 and March 2017 were identified by a search strategy. Excluded those with bad images or categorized as neither PR nor PE, totally 872 patients with PR (n = 656) and PE (n = 216) were identified and divided into two groups according to optical coherence tomography (OCT) imaging. The definition of STEMI and identification of the culprit lesion were described previously [8] and identification of PR and PE was based on established OCT diagnostic criteria [11]. OCT-rupture was defined and categorized according to the imaging of fibrous cap disruption and the presence of thrombus, OCT-erosion was defined and categorized according to the absence of fibrous cap disruption and the presence of thrombus. The optimal medical therapy for STEMI was administered in every patient during hospitalization, according to the current guidelines.

### Data collection

CHD risk factors of interest included age, gender, hypertension, DM history, hyperlipemia, AMI history and current smoking. All individuals had a 12-lead or 18-lead electrocardiogram measured and left ventricular ejection fraction (LVEF) was measured within 24 h of admission by an experienced practitioner using Simpson’s biplane method of disks. Blood glucose-related variables (ARPG, PFPG, and HbA1c), complete blood count, electrolytes, creatinine, lipid parameters, hypersensitive C-reactive protein (hs-CRP), and maximum Cardiac Troponin I (cTnI), as well as the data of coronary angiography and OCT images, were collected.

**Fig. 1:**
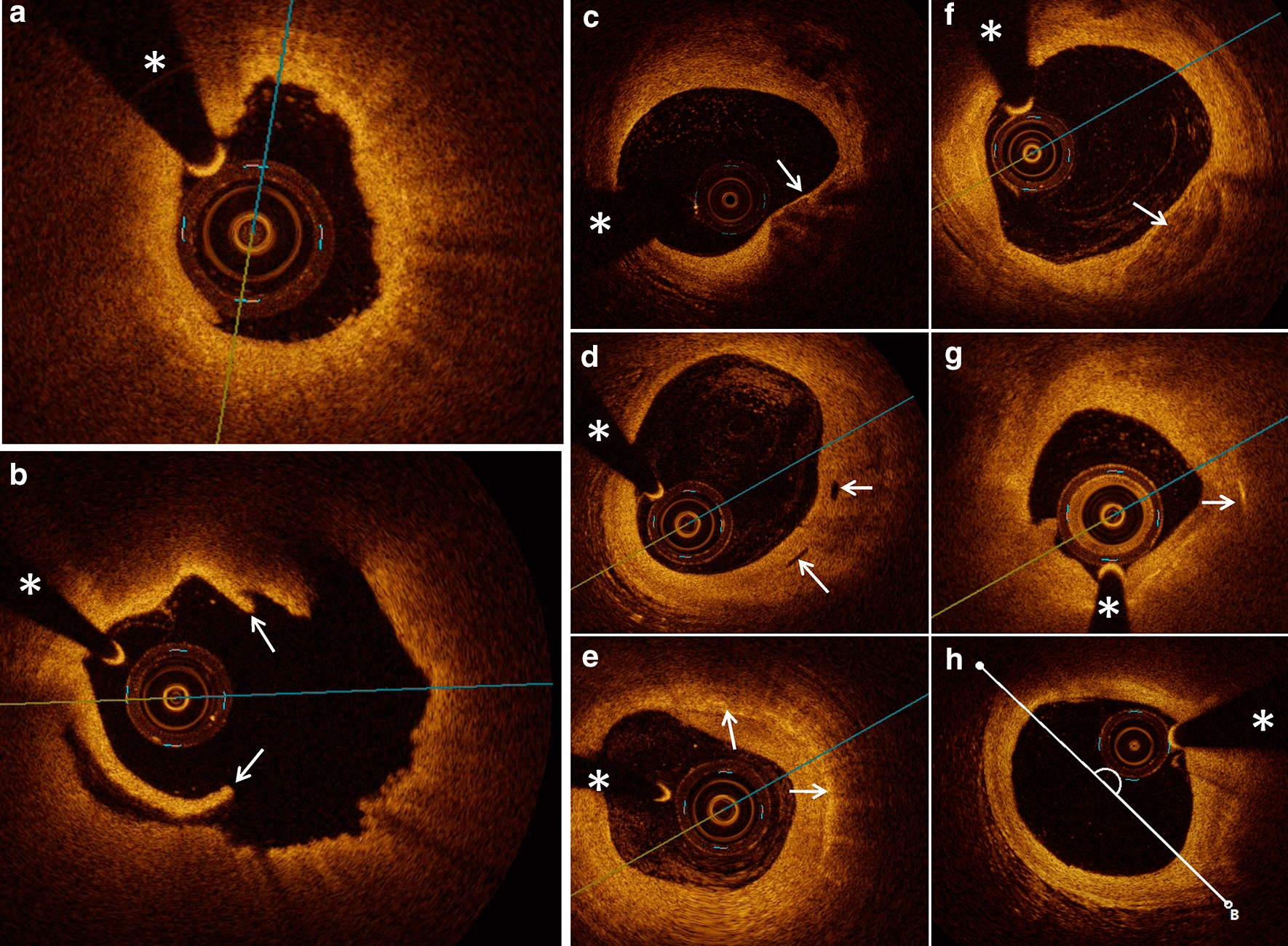
Typical optical coherence tomography (OCT) images of plaque characteristics. **a** A typical image of a patient with STEMI and plaque erosion characterized as intact and rouHbA1c intima attached with small white thrombus. **b** A typical image of a patient with STEMI and plaque rupture characterized as ruptured intima (arrow) backed with a big cavity. **c**–**h** Representative OCT images, including TCFA, micro-channel, macrophage accumulation, spotty calcification, cholesterol crystal, and lipid core measured with the lipid arc, are presented in turn (arrows). The asterisk marks the position of the OCT guide wire

Quantitative coronary angiography (QCA) was performed using the Cardiovascular Angiography Analysis System (CAAS, 5.10, Pie Medical Imaging B.V., Maastricht, the Netherlands). Culprit vessels and lesion sites were identified, and the length of the culprit lesions, reference vessel diameter, minimal lesion diameter, and degree of diameter stenosis were measured. OCT data were recorded using the C7-XR OCT intravascular imaging system (OCT C7 Dragonfly, St. Jude Medical, St. Paul, MN, USA). Quantitative and qualitative analyses of underlying plaques were performed by two independent operators as described in a previous study [10]. Any discordance was resolved by consensus with a third reviewer. Using established diagnostic criteria [12], the plaque and thrombus type were identified based on established OCT diagnostic criteria, along with identification of microchannels, cholesterol crystals, macrophages, and thin-cap fibroatheroma (TCFA). The lesion length, minimal lumen area, minimal thickness of the fibrotic cap, length of the residual thrombus, length of the lipid core, maximal arc of the lipid core, and average arc of the lipid core were measured as described previously [13] (Fig. 1)

### Statistical analysis

Dichotomous variables were expressed as n (%) and continuous variables as mean ± SD or median (P25, P75), with normality of variables determined by the Kolmogorov–Smirnov test. For categorical data, Chi-squared or Fisher’s exact test was used, with comparisons of continuous data done by Student’s t test for normal variables or the Mann–Whitney test for non-Gaussian variables. Inter- and intra-observer reliability was assessed by Kappa statistics. A two-tailed P-value < 0.05 was considered statistically significant.

Patients were divided into four groups according to their blood ARPG levels: non AH group [ARPG < 6.1 mmol/L (< 110 mg/dL)]; mild AH group [6.1–6.9 mmol/L (110–125 mg/dL)]; moderate AH group [7.0–11.0 mmol/L (126–198 mg/dL)] and severe AH group [> 11.0 mmol/L (> 198 mg/dL)]. PFPG levels were stratified into four groups as follow: group I [PFPG < 5.6 mmol/L (< 100 mg/dL)]; group II [5.6–6.0 mmol/L (100–110 mg/dL)]; and group III [6.1–6.9 mmol/L (110–126 mg/dL)] and group IV [> 7.0 mmol/L (> 126 mg/dL)]. Patients were also divided into two groups according to their blood HbA1c levels: low HbA1c group (HbA1c < 6.5%) and high HbA1c group (HbA1c ≥ 6.5%). GVT was defined as the ARPG-to-PFPG ratio and grouped as GVT-normal group (ARPG < 6.1 mmol/L and PFPG < 5.6 mmol/L), GVT-up group (ARPG ≥ 6.1 mmol/L and/or PFPG ≥ 5.6 mmol/L, and ARPG/PFPG < 1), and GVT-down group (ARPG ≥ 6.1 mmol/L and/or PFPG ≥ 5.6 mmol/L, and ARPG/PFPG > 1). Chronic plasma glucose (CPG) was calculated by formula (CPG (mg/dL) = 28.7 × HbA1c-46.7). A/C was defined as the ARPG-to-CPG ratio (Table 2).

Univariate and multivariate logistic regression analyses were applied to screen the glucose-related variables that were significantly associated with the identification of plaque type. A P < 0.05 denoted statistical significance. Significant variables were subsequently included in a differential diagnostic model to evaluate the diagnosis value. Furthermore, the correlation between abnormal blood glucose and plaque morphology was analyzed with Spearman correlation. These analyses were all produced by EmpowerStats (www.empowerstats.com.) using R.

## Results

Baseline characteristics between PR and PE are shown as Additional file 1: Table S1. Age (P < 0.001), men gender (P = 0.019), current smoking (P = 0.05), history of hypertension (HTN) (P = 0.001), low-density lipoprotein cholesterol levels (LDL-C) (P < 0.001), triglyceride levels (TC) (P = 0.012). Angiography imaging data between PR and PE are shown as Additional file 1: Table S1. The inter-observer kappa coefficients for plaque rupture and plaque erosion were 0.866 and 0.870, respectively. The intra-observer kappa coefficients for plaque rupture and plaque erosion were 0.891 and 0.905, respectively. Initial TIMI flow (P = 0.003); culprit vessel (P < 0.001), multivessel lesion (P < 0.001), stenosis degree (P < 0.001) and length of culprit lesion (P = 0.002) showed significant difference between groups. The results of the study were consistent with the results of our previous study [10]. The glucose-related variables between PR and PE are presented as Table 1. As a result, all the glucose-related variables except PFPG presented significant difference between PR and PE.

**Table 1:** Glucose-related variables between plaque rupture and plaque erosion

Glucose-related variables	Plaque rupture	Plaque erosion	*P*-value
	N = 656	N = 216	
DM history	192 (29.27%)	46 (21.30%)	0.023
HbA1c (%)	5.90 (5.60–7.00)	5.70 (5.40–6.30)	0.002
CG (mmol/L)	6.81 (6.33–8.57)	6.49 (6.02–7.45)	0.002
ARPG (mmol/L)	8.39 (6.92–10.98)	7.54 (6.29–9.55)	< 0.001
PFPG (mmol/L)	6.93 (5.54–9.36)	6.38 (5.43–8.48)	0.072
A/C glycemic ratio	1.21 (1.05–1.41)	1.14 (0.97–1.33)	0.013
GVT			0.002
Normal	26 (4.56%)	23 (11.92%)	
Up	160 (28.07%)	50 (25.91%)	
Down	384 (67.37%)	120 (62.18%)	

Data are presented as mean ± SD or Median (Q1–Q3)/N (%)

*ARPG* admission plasma glucose, *A/C* admission/chronic glycemic ratio, *CG* chronic plasma glucose, *DM* diabetes mellitus, *GVT* glucose variable tendency, *HbA1c* glycosylated hemoglobin, *PCI* percutaneous coronary intervention, *PFPG* post-PCI fasting plasma glucose

Logistic regression analysis showed positive and significant association between plaque rupture and the glucose-related variables included HbA1c, ARPG, DM history, and GVT. Yet PFPG and A/C showed no significant difference between the groups. After adjustment by the risk factors variables associated with plaque model, including age, gender, HTN history, current smoking, and LDL-C levels, only the difference of ARPG levels and GVT remained significantly between the groups. Added initial TIMI flow, culprit vessel and multi-vessel lesion into the adjustment variables, the difference of ARPG levels and GVT between PR and PE did not disappear either. Stratification analysis showed significant difference of both moderate and severe AH groups between groups no matter whether adjusted or not. The higher the ARPG levels of the patient, the greater the risk of PR (Table 2).

**Table 2:** Logistic regression analysis of glucose-related variables between plaque rupture and erosion

Exposure	Statistics	Non-adjusted	Adjustment I	Adjustment II
HbA1c (%)	6.43 ± 1.44	1.19 (1.03, 1.36) 0.0153	1.13 (0.98, 1.30) 0.0915	1.12 (0.97, 1.30) 0.1264
ARPG (mmol/L)	9.30 ± 3.80	1.08 (1.03, 1.13) 0.0018	1.06 (1.01, 1.12) 0.0141	1.06 (1.01, 1.11) 0.0256
PFPG (mmol/L)	7.95 ± 3.56	1.03 (0.98, 1.08) 0.2441	1.01 (0.96, 1.06) 0.7984	1.01 (0.96, 1.06) 0.8090
A/C	1.25 ± 0.37	1.60 (0.94, 2.73) 0.0858	1.46 (0.85, 2.51) 0.1714	1.43 (0.83, 2.45) 0.1943
DM history				
No	634 (72.7%)	1	1	1
Yes	238 (27.3%)	1.53 (1.06, 2.21) 0.0231	1.42 (0.96, 2.09) 0.0800	1.38 (0.92, 2.05) 0.1155
GVT				
Normal	49 (6.4%)	1	1	1
Up	210 (27.5%)	2.83 (1.48, 5.39) 0.002	2.01 (1.02, 3.99) 0.045	2.03 (1.02, 4.07) 0.044
Down	504 (66.1%)	2.83 (1.56, 5.14) 0.001	2.16 (1.15, 4.04) 0.016	2.04 (1.09, 3.85) 0.027
Strati-HbA1c				
< 6.5	471 (70.2%)	1	1	1
≥ 6.5	200 (29.8%)	1.18 (0.45, 3.12) 0.7393	1.29 (0.47, 3.54) 0.6234	1.34 (0.48, 3.76) 0.5778
Strati-ARPG				
< 6.1	117 (13.4%)	1	1	1
≥ 6.1, < 7.1	144 (16.5%)	1.41 (0.84, 2.37) 0.1909	1.25 (0.71, 2.20) 0.4396	1.17 (0.66, 2.08) 0.5945
≥ 7.1, < 11.1	412 (47.3%)	2.07 (1.32, 3.24) 0.0014	1.63 (1.00, 2.67) 0.0517	1.58 (0.96, 2.61) 0.0736
≥ 11.1	199 (22.8%)	2.29 (1.24, 4.21) 0.0080	2.05 (1.05, 3.99) 0.0345	1.99 (1.02, 3.90) 0.0438
Strati-PFPG				
< 5.6	204 (26.7%)	1	1	1
≥ 5.6, < 6.1	88 (11.5%)	0.92 (0.53, 1.60) 0.7804	0.81 (0.46, 1.44) 0.4826	0.87 (0.49, 1.56) 0.6417
≥ 6.1, < 7.0	112 (14.7%)	1.01 (0.61, 1.70) 0.9603	0.90 (0.52, 1.53) 0.6905	0.90 (0.52, 1.55) 0.7027
≥ 7.0	359 (47.1%)	1.37 (0.93, 2.04) 0.1145	1.10 (0.72, 1.67) 0.6527	1.14 (0.74, 1.74) 0.5611

Data are presented as OR (95% CI) P value. Adjustment I for: age; gender; HTN history; current smoking; LDL-C. Adjustment II for: age; gender; HTN history; current smoking; LDL-C; initial TIMI flow; multivessel lesion, and culprit vessel

*A/C* admission/chronic glycaemia ratio, *ARPG* admission random plasma glucose, *DM* diabetes mellitus, *GVT* glucose variable tendency, *HbA1c* glycosylated hemoglobin, *HTN* hypertension, *LDL-C* low density lipoprotein cholesterol, *PCI* percutaneous coronary intervention, *PFPG* post-PCI fasting plasma glucose, *Strati-* stratification, *TIMI* thrombolysis in myocardial infarction

The association between the glucose variables and PR risk was then analysed in patients with or without DM history (Table 3). Logistic regression analysis showed positive and significant association of GVT and continuous ARPG with PR risk only in nondiabetic patients. Among the nondiabetic patients, stratification analysis showed that only moderate AH was positively and significantly associated with PR risk. After adjustment by the risk factors variables associated with plaque model, including age, gender, HTN history, current smoking, and LDL-C levels, the association weakened. Added initial TIMI flow, culprit vessel and multi-vessel lesion into the adjustment variables, the predictive ability for PR risk of GVT reserved but ARPG vanished. Given without enough patients, stratified ARPG of patients with DM history were failed to be analysed.

**Table 3:** Logistic regression analysis of AG between plaque rupture and erosion with or without DM history

Exposure	NDM history	DM history	Total
Crude			
Continuous HbA1c	1.62 (0.87, 3.02) 0.1303	1.04 (0.84, 1.30) 0.6970	1.10 (0.89, 1.36) 0.3874
Continuous ARPG	1.11 (1.02, 1.21) 0.0182	1.04 (0.96, 1.12) 0.3539	1.07 (1.01, 1.14) 0.0213
Continuous PFPG	1.04 (0.94, 1.14) 0.4550	0.96 (0.89, 1.05) 0.3658	0.99 (0.93, 1.06) 0.8630
Continuous A/C	1.56 (0.80, 3.08) 0.1930	1.36 (0.57, 3.28) 0.4880	1.59 (0.57, 3.28) 0.0860
GVT			
Normal	1		
Up	2.79 (1.40, 5.55) 0.0030	1	
Down	2.54 (1.38, 4.67) 0.0030	1.32 (0.67, 2.59) 0.4170	
Stratified ARPG			
< 6.1	1	1	1
≥ 6.1, < 7.0	1.53 (0.91, 2.59) 0.1107	–	1.41 (0.84, 2.37) 0.1909
≥ 7.0, < 11.1	2.14 (1.36, 3.38) 0.0011	–	2.07 (1.32, 3.24) 0.0014
≥ 11.1	1.96 (0.93, 4.15) 0.0781	–	2.29 (1.24, 4.21) 0.0080
Stratified PFPG			
< 5.6	1	1	1
≥ 5.6, < 6.1	0.86 (0.50, 1.51) 0.6089	–	0.92 (0.53, 1.59) 0.7621
≥ 6.1, < 7.0	1.06 (0.62, 1.80) 0.8403	0.40 (0.03, 5.15) 0.4822	1.00 (0.60, 1.68) 0.9981
≥ 7.0	1.25 (0.77, 2.01) 0.3685	0.78 (0.09, 6.87) 0.8232	1.20 (0.76, 1.90) 0.4250
Adjust I			
Continuous ARPG	1.07 (0.97, 1.18) 0.1528	1.06 (0.98, 1.16) 0.1609	1.06 (1.00, 1.13) 0.0710
Stratified ARPG			
< 6.1	1	1	1
≥ 6.1, < 7.0	1.38 (0.77, 2.45) 0.2756	–	1.25 (0.71, 2.20) 0.4396
≥ 7.0, < 11.1	1.72 (1.04, 2.85) 0.0347	–	1.63 (1.00, 2.67) 0.0517
≥ 11.1	1.58 (0.70, 3.60) 0.2709	–	2.05 (1.05, 3.99) 0.0345
GVT			
Normal	1		
Up	2.12 (1.02, 4.41) 0.0450		
Down	1.95 (1.03, 3.69) 0.0410		
Adjust II			
Continuous ARPG	1.07 (0.98, 1.18) 0.1408	1.05 (0.96, 1.15) 0.2630	1.05 (0.99, 1.12) 0.0983
Stratified ARPG			
< 6.1	1	1	1
≥ 6.1, < 7.0	1.29 (0.72, 2.33) 0.3920	–	1.17 (0.66, 2.08) 0.5945
≥ 7.0, < 11.1	1.67 (1.00, 2.80) 0.0514	–	1.58 (0.96, 2.61) 0.0736
≥ 11.1	1.67 (0.72, 3.85) 0.2313	–	1.99 (1.02, 3.90) 0.0438
GVT			
Normal	1		
Up	2.16 (1.03, 4.55) 0.0420		
Down	1.89 (0.99, 3.60) 0.0550		

Data are presented as OR (95% CI) P value. Adjustment I for: age; gender; HTN history; current smoking; LDL-C. Adjustment II for: age; gender; HTN history; current smoking; LDL-C; initial TIMI flow; multivessel lesion, and culprit vessel

*A/C* admission/chronic plasma glucose, *ARPG* admission random plasma glucose, *DM* diabetes mellitus, *GVT* glucose variable tendency, *HbA1c* glycosylated hemoglobin, *HTN* hypertension, *LDL-C* low density lipoprotein cholesterol, *NDM* nondiabetic mellitus, *PCI* percutaneous coronary intervention, *PFPG* post-PCI fasting plasma glucose, *TIMI* thrombolysis in myocardial infarction

Subsequently, we quantified the diagnostic accuracy of AG and GVT for PR using the receiver operating characteristic curve (ROC). Preliminary analysis of ROC indicated that the area under curve (AUC) of ARPG was larger than that of GVT. Combined ARPG or GVT with the risk factors (age, gender, smoking, hypertension, and LDL-C) to establish combined predictive models successively, the predictive efficiency and accuracy increased significantly. Added initial TIMI flow, culprit vessel and multi-vessel lesion into the combined model, the predictive ability increased more and to over 70% (AUC = 0.7087, 0.7022; specificity = 63.82%, 57.07%; sensitivity = 69.83%, 73.53%; and accuracy = 68.29%, 69.36%; respectively) (Additional file 1: Table S2).

Besides, the correlations between abnormal hyperglycemia variables and data of angiography and OCT were analyzed using Spearman’s correlation. As a result, ARPG, PFPG and HbA1c were determined to be positively and weakly correlated with the mean size of lipid arc. And HbA1c was also positively and weakly correlated with TCFA and maximal lipid arc (Additional file 1: Table S3). Further analysis was performed on the association between stratified ARPG and imaging characteristics of STEMI patients using logistic regression. The results indicated that the risk of PR increased as ARPG levels rose (P < 0.001). And the higher the ARPG level was, the higher risk of TCFA (P = 0.007) and the bigger size of lipid arc was (Table 4).

**Table 4:** QCA and OCT characteristics among ARPG groups

ARPG group (mmol/L)	< 6.1	≥ 6.1, < 7.0	≥ 7.1, < 11.1	≥ 11.1
	N = 117	N = 144	N = 412	N = 199
QCA				
MLD (mm)	0.79 (0.64–1.08)	0.83 (0.66–1.10)	0.87 (0.65–1.10)	0.78 (0.62–0.99)
RVD (mm)	2.69 ± 0.58	2.69 ± 0.51	2.70 ± 0.58	2.59 ± 0.50
DS (%)	69.00 (61.00–76.00)	68.00 (61.00–74.00)	67.00 (60.00–75.00)	69.00 (63.00–75.00)
LL (mm)	22.39 ± 8.69	21.21 ± 8.18	21.73 ± 8.42	22.36 ± 11.26
OCT				
Plaque rupture*	73 (62.39%)	101 (70.14%)	321 (77.91%)	161 (80.90%)
Culprit vessel				
RCA	34 (29.06%)	61 (42.36%)	171 (41.50%)	77 (38.69%)
LAD	66 (56.41%)	70 (48.61%)	211 (51.21%)	102 (51.26%)
LCX	17 (14.53%)	13 (9.03%)	30 (7.28%)	20 (10.05%)
Culprit site				
Proximal	41 (35.04%)	55 (38.19%)	189 (45.87%)	90 (45.23%)
Middle	54 (46.15%)	56 (38.89%)	160 (38.83%)	68 (34.17%)
Distal	22 (18.80%)	33 (22.92%)	63 (15.29%)	41 (20.60%)
Multivessel lesion	67 (57.26%)	93 (64.58%)	279 (67.72%)	137 (68.84%)
Lesion length	16.84 ± 6.44	17.49 ± 5.95	16.93 ± 6.53	17.51 ± 6.75
Lipid length*	8.95 ± 6.11	10.67 ± 6.04	9.83 ± 6.52	10.88 ± 6.29
Mean lipid arc*	170.45 (0.00–235.54)	196.78 (0.00–253.35)	188.55 (0.00–245.77)	210.66 (158.92–257.20)
Max lipid arc*	222.10 (0.00–360.00)	299.60 (0.00–360.00)	290.65 (0.00–360.00)	324.60 (212.95–360.00)
Minimal FCT	0.02 (0.00–0.05)	0.03 (0.00–0.04)	0.03 (0.00–0.04)	0.03 (0.02–0.04)
TCFA*	62 (52.99%)	86 (60.99%)	238 (58.33%)	140 (70.35%)
Chole- crystal	43 (36.75%)	63 (44.68%)	172 (42.16%)	89 (44.95%)
Microchannel	40 (34.19%)	34 (24.11%)	113 (27.70%)	53 (26.63%)
Thrombus length	4.80 (2.70–7.90)	5.50 (3.40–8.90)	5.10 (3.00–7.88)	5.50 (2.90–8.78)
Thrombus type				
None	9 (7.69%)	12 (8.45%)	38 (9.27%)	20 (10.10%)
White	55 (47.01%)	73 (51.41%)	214 (52.20%)	84 (42.21%)
Red	2 (1.71%)	5 (3.52%)	10 (2.44%)	1 (0.50%)
Mix	51 (43.59%)	52 (36.62%)	148 (36.10%)	93 (46.73%)

Data presented as Mean (SD) Median (Q1–Q3)/N (%)

*ARPG* admission random plasma glucose, *Chole*-*crystal* cholesterol crystal, *DS* the degree of stenosis, *FCT* thickness of fibrotic cap, *LAD* left anterior descending branch, *LCX* left circumflex branch, *LL* lesion length, *MLD* minimal lumen diameter, *OCT* optical coherence tomography, *QCA* quantitative coronary angiography, *RCA* right coronary artery, *RVD* reference vessel diameter, *TCFA* thin-cap fibroatheroma

*P value < 0.05

## Discussion

To the best of our knowledge, present study is the first one to compare the association of glucose-related variables with plaque rupture and plaque erosion identified by OCT in STEMI patients. The findings are as showed below: (1) HbA1c, ARPG, GVT, A/C, and DM history were significantly and positively associated with PR risk in STEMI patients before adjustment for traditional risk factors; (2) Among the glucose-related variables, ARPG and GVT were independent predictors of PR in STEMI patients without DM rather than in those with DM; (3) The higher the ARPG levels of the STEMI patient, the greater the risk of PR; (4) High ARPG and HbA1c were positively related with plaque vulnerability; (5) The higher the ARPG levels of the STEMI patient, the higher risk of TCFA and the bigger size of lipid arc among ARPG groups.

The definite DM history has immediate association with worse cardiovascular outcomes [14]. The relationship between plaque rupture and DM was well described in the previous studies [15], but the relationship between plaque erosion and glucose condition is still unclear. High ARPG levels after AMI are common and play a role in predicting adverse events in DM and non-diabetic patients [2, 16]. Especially during hospitalization, it is a strong predictable factors for fatal outcomes in patients with AMI [5, 17]. But A/C glycemic ratio was reported to be a better predictor of in-hospital morbidity and mortality than glycaemia at admission [3]. And positive correlation of serum HbA1c with the presence of carotid plaque [1] and vulnerable plaque in ACS were reported in previous studies [16]. In present study, we compared the association and predictive ability of ARPG, HbA1c, A/C and DM history with PR and PE. Compared with PE, PR was more related with the glucose-related variables before adjustment with the traditional risks. And the association between ARPG and PR was confirmed after the adjustment. Therefore ARPG was identified as an independent predictor for PR in STEMI patients. Although the predictive ability was not strong, it raised an auxiliary factor for distinguishing PR and PE in STEMI patients clinically. It also induced our deep thought on the mechanism of the onset and development of PR and PE.

Previous studies indicated that patients with AMI caused by plaque rupture had some clinical characteristics [7] same as those with admission hyperglycemia did, such as more multivessel lesions [18], more no-reflow occurrence during PCI [19], larger infarct sizes than normoglycemic patients [20], and accompanied by long term [21] and short term poor outcomes [22]. Our results provided a strong clinical evidence for the closer association of AH and STEMI patients with PR than PE. The higher the ARPG, the greater the PR risk a STEMI patient has. Admission hyperglycemia in STEMI patients might play a role in coronary thrombosis through altering clot features and enhancing local thrombin generation and platelet activation [23]. It is associated with the large thrombus burden which causes adverse cardiac outcomes [24]. There were no relationships of ARPG to thrombus length and type in the present study which might due to that the thrombus overlying the lesion might have been dissolved before OCT imaging [11]. We guess the treatment targeting AH such as intensified insulin-based glycaemia control may benefit STEMI patients with PR via reducing thrombosis and thrombus burden. It can also explain why the intensified insulin-based glycaemia control after the onset of STEMI might improve the prognosis [25]. And the intensified insulin-based glycaemia control should get more attention in the patients with STEMI caused by PR than those by PE.

In addition, present study only indicated the association of AH with PR in STEMI patients without DM but not those with DM. Elevated ARPG in nondiabetic patients may link to impaired glucose tolerance (IGT) which was more associated with PR than normal glucose tolerance (NGT) [26]. On the other hand, although ARPG in nondiabetic AMI patients could offer an initial screening tool for those patients with high risk for future DM [27], it does not represent previously undiagnosed DM and abnormal glucose tolerance [28]. The elevated ARPG levels in nondiabetic patients with STEMI patients are independently associated with larger infarct size and higher long-term mortality rates [29]. An elevated ARPG level not only disturbed glucose metabolism that worsens the prognosis, but also accompanied by elevated stress hormone and reflected acute stress state [30, 31]. Stress hyperglycaemia with myocardial infarction is associated with a poor prognosis in DM and nondiabetic patients [32, 33] via disturbing myocardial blood flow and energetics, contributing to high monocyte chemo-attractant protein-1 levels and sFas apoptosis levels [34, 35], inducing a pro-oxidative/pro-inflammatory state [36, 37], and resulting in coronary plaque rupture eventually [38]. It might partly explain why the patients with AH continue to have a higher mortality even after rapid revascularization and treatment of hyperglycaemia [18]. Considering of the closer association between ARPG and PR than PE resulted from present study, treatment of antioxidant and antagonistic stress should benefit more and be taken more seriously in the STEMI patients with PR than in those with PE.

Although no association between HbA1c and plaque morphology in STEMI patients was found by multivariable logistic regression analysis after adjustment for traditional risk factors, both HbA1c and ARPG were determined to be positively correlated with degree of lumen stenosis and the lipid content in the plaque on the evidence of imaging, and HbA1c seemed have more correlation than ARPG. It provided a clinical evidence for the role chronic hyperglycemia might play in the developing of vulnerable plaque which characterized with large lipid core. It may partly explain why patients with DM had a higher prevalence of lipid-rich plaque but similar prevalence of plaque rupture and plaque erosion was also been detected in a study by Tomoyo Sugiyama [15]. This also helps explain why increased duration of DM combined with higher HbA1c levels is associated with negative coronary artery remodeling [39] and influences culprit-plaque characteristics in patients with AMI [40]. High HbA1c levels may partly result from insulin resistance (IR) that subsequently increased spotty calcification and plaque vulnerability [41]. And the increased spotty calcification led to large intrinsic calcification angle (ICA) that involved in the formation of vulnerable plaques that eventually lead to plaque rupture [42]. The effective control of glucose chronically before the onset of STEMI caused by PR might benefit DM patients via improving impaired beta cell function and glycometabolic derangements [43] and prevent them from significant and insidious coronary atherosclerosis which developed vulnerable plaque and subsequent high risk for plaque rupture and cardiac events [44]. And the lipid regulation therapy might benefit either STEMI patients with AH levels or those with high HbA1c, the latter seemed more than the former.

Besides, dynamic glucose fluctuation may be associated with PR via increasing CD14(bright)CD16(+) monocyte levels preferentially [38]. Considering glucose variability was reported to be correlated with PR in previous studies, we analyzed glucose variable tendency (GVT) to value it between PR and PE in present study too. Compared to PE, positive and significant association was showed between GVT and PR as well, though GVT defined as the ratio of PFPG/ARPG in present study may not represent the dynamic glucose fluctuation completely. It may not only provide a risk factor in identifying PE or PR in STEMI patients, but also helps to simplify and facilitate clinical monitoring and prognosis stratification.

Previous studies also indicated that coronary plaques in CAD patients with NGT are more stable than in those with IGT and DM [45]. However, no correlation between PFPG with plaque morphological characteristics was identified in present study. It might due to the population studied herein was enrolled from only STEMI patients whose coronary atherosclerosis developed significantly no matter caused by PR or PE. And PFPG which was interfered by acute stress and in-hospital treatment seemed unsuitable to be a variable for diagnosing IGT.

## Study limitations

There are several limitations of this study. First, it’s a single center observational study based on retrospective design. In the present study, a total of 233/1005 cases were excluded due to poor quality of OCT image or identified as neither PR nor PE, therefore some patients with STEMI caused by PR and PE were failed to be included and those patients with STEMI caused by other reasons include calcified nodules were not studied. Second, the plasma glucose was not monitored after the acute event so that mean amplitude of glycemic excursion and stress hyperglycemia failed to be studied. Last, an important risk factor body mass index (BMI) has not been adjusted because of incomplete weiHbA1ct and heiHbA1ct record. Being a subanalysis of the EROSION study, present study was a retrospective study focus on cardiovascular specialty, and other glucose-related variables (such as DM duration, the kind of diabetic drugs, IRI level, and HOMA-R) were failed to have been collected.

## Conclusions

The findings of present study highlight the association of ARPG and GVT with plaque rupture in STEMI patients, especially in patients with admission glucose levels ≥ 7.1 mmol/l. ARPG and GVT are independent predictors for PR in STEMI patients without DM. And high HbA1c and ARPG were positively correlated with the development of vulnerable plaque in culprit vessels, the former more than the latter.

## Supplementary information

**Additional file 1: Table S1.** Baseline characteristics between plaque rupture and plaque erosion. **Table S2.** Presictive models of PR established using ARPG/GVT single or combined with traditional risk factors. **Table S3.** The correlation between glucose-related variables and plaque morphological characteristics using Spearman correlation.

## Data Availability

The datasets used and analyzed during the present study are available from the corresponding author on reasonable request.

## Abbreviations

ARPG: Random plasma glucose on admission
AH: Admission hyperglycemia
AMI: Acute myocardial infarction
A/C: Acute-to-chronic glycemic ratio
AUC: Area under curve
CPG: Chronic plasma glucose
CHD: Coronary heart disease
CK-MB: Creatine kinase-MB
cTnI: Cardiac troponin
DM: Diabetes mellitus
HbA1c: Glycosylated hemoglobin
hs-CRP: Highly sensitive C-reactive protein
LDL: Low-density lipoprotein cholesterol levels
LVEF: Left ventricular ejection fraction
OCT: Optical coherence tomography
PCI: Percutaneous coronary intervention
PE: Plaque erosion
PFPG: Post-PCI fasting plasma glucose
PR: Plaque rupture
proBNP: Pro-brain natriuretic peptide
QCA: Quantitative coronary angiography
ROC: Receiver operating characteristic curve
STEMI: ST-segment elevation myocardial infarction
TCFA: Thin-cap fibroatheroma
TIMI: Thrombolysis in myocardial infarction
