# Supplementary material for: The association between glucose-related variables and plaque morphology in patients with ST-segment elevated myocardial infarction
Source: Cardiovasc Diabetol. 2020 Jul 8;19:109. doi: 10.1186/s12933-020-01074-9 (PMC7341636; doi:10.1186/s12933-020-01074-9)
Supplement: Supplementary file 1 — Additional file 1: Table S1. Baseline characteristics between plaque rupture and plaque erosion. Table S2. Presictive models of PR established using ARPG/GVT single or combined with traditional risk factors. Table S3. The correlation between glucose-related variables and plaque morphological characteristics using Spearman correlation. [file 12933_2020_1074_MOESM1_ESM.docx]

**Additional material**

**The Association between Glucose-related Variables and**

**Plaque Morphology in Patients with ST-segment Elevated Myocardial Infarction**

**Additional file 1**

TableS1-S3

| **TableS1. Baseline characteristics between plaque rupture and plaque erosion** | | | |
| --- | --- | --- | --- |
| Variables | Plaque rupture | Plaque erosion | *P*-value |
|  | N=656 | N=216 |  |
| Men | 459 (69.97%) | 169 (78.24%) | 0.019 |
| Age(years) | 58.75±10.71 | 54.71±10.40 | <0.001 |
| Age>50year | 503 (76.68%) | 140 (64.81%) | <0.001 |
| Current smoking | 339 (51.68%) | 132 (61.11%) | 0.05 |
| HTN history | 321 (48.83%) | 86 (39.81%) | 0.001 |
| AMI history | 22 (3.35%) | 5 (2.31%) | 0.65 |
| LVEF (%) | 60.00 (55.00-62.00) | 60.00 (55.00-62.00) | 0.842 |
| Lipid profile |  |  |  |
| TG(mg/dl) | 129.32 (93.87-179.33) | 120.42 (85.47-171.83) | 0.195 |
| TC(mg/dl) | 185.42 (160.87-211.23) | 171.89 (146.37-210.56) | 0.012 |
| HDL-C(mg/dl) | 47.95 (41.67-56.46) | 47.56 (40.80-56.26) | 0.755 |
| LDL-C(mg/dl) | 118.64 (101.42-145.88) | 108.66 ( 86.43-139.34) | <0.001 |
| other laboratory data |  |  |  |
| Creatinine (umol/L) | 76.45 (63.80-90.70) | 73.60 (62.68-86.00) | 0.065 |
| Troponin I(μg/L) | 1.31 (0.24-6.98) | 1.18 (0.19-10.61) | 0.982 |
| pro-BNP(pg/ml) | 602.00 (111.50-1486.00) | 584.50 ( 91.50-1447.25) | 0.694 |
| hs-CRP(mg/L) | 5.97 (2.30-12.53) | 5.11 (2.25-11.98) | 0.423 |
| Multivessel lesion | 457 (69.66%) | 119 (55.09%) | <0.001 |
| Initial TIMI flow(=0) | 647 (98.63%) | 205 (94.91%) | 0.003 |
| Culprit vessel |  |  | <0.001 |
| RCA | 284 (43.29%) | 59 (27.31%) |  |
| LAD | 310 (47.26%) | 139 (64.35%) |  |
| LCX | 62 (9.45%) | 18 (8.33%) |  |
| Culprit site |  |  | 0.623 |
| proximal | 99 (45.83%) | 276 (42.07%) |  |
| middle | 80 (37.04%) | 258 (39.33%) |  |
| distal | 37 (17.13%) | 122 (18.60%) |  |
| QCA data |  |  |  |
| MLD | 0.79 (0.61-1.04) | 0.94 (0.75-1.17) | <0.001 |
| RVD | 2.66±0.55 | 2.72±0.57 | 0.121 |
| Stenosis degree (%) | 70.00 (62.00-76.00) | 64.00 (58.00-69.00) | <0.001 |
| Lesion length | 21.35 (16.32-26.91) | 18.80 (15.19-23.22) | 0.002 |
| Pre-medicate |  |  |  |
| DAPT | 656 (100%) | 216 (100%) | - |
| Statins | 637 (97.10%) | 211 (97.69%) | 0.812 |
| Beta-blockers | 348 (53.05%) | 134 (62.04%) | 0.021 |
| CCB | 38 (5.79%) | 12 (5.56%) | 0.897 |
| ACEI or ARB | 408 (62.20%) | 140 (64.81%) | 0.49 |
| Data are presented as mean ±SD or Median (Q1-Q3)/ N(%).Abbreviations: ACEI, angiotensin converting enzyme inhibitor; AMI, acute myocardial infarction; ARB, Angiotensin receptor blocker; CCB, calcium channel blocker; DAPT, double antiplatelet therapy; hs-CRP, hypersensitive C-reactive protein; HTN, hypertension; HDL-C, high-density lipoprotein cholesterol; LAD, Left anterior descending branch; LCX, left circumflex branch; LDL-C, low-density lipoprotein cholesterol; LVEF, left ventricular ejection fraction; MLD, minimum lumen diameter; pro-BNP, brain natriuretic peptide precursor; QCA, quantitative coronary angiography; RCA, Right coronary artery; RVD, reference vessel diameter; TC, total cholesterol; TG, triglyceride; TIMI, thrombolysis in myocardial infarction; . | | | |

| **TableS2:Presictive models of PR established using ARPG/GVT single or combined with traditional risk factors** | | | | | | |
| --- | --- | --- | --- | --- | --- | --- |
| Parameters | ARPG | GVT | combination Ⅰ | | combination Ⅱ | |
|  |  |  | ARPG | GVT | ARPG | GVT |
| PR(n) | 656 | 582 | 580 | 563 | 580 | 563 |
| PE(n) | 216 | 197 | 199 | 191 | 199 | 191 |
| ROC-AUC | 0.592 | 0.533 | 0.6814 | 0.6761 | 0.7087 | 0.7022 |
| 95%CI lower | 0.5477 | 0.4922 | 0.6393 | 0.6332 | 0.6663 | 0.659 |
| 95%CI upper | 0.6363 | 0.5739 | 0.7234 | 0.7189 | 0.7511 | 0.7455 |
| best threshold | 1.0136 | 1.0802 | 1.2533 | 1.0981 | 0.9206 | 0.8836 |
| Specificity | 0.5509 | 0.4264 | 0.7638 | 0.6649 | 0.6382 | 0.5707 |
| Sensitivity | 0.5991 | 0.634 | 0.5207 | 0.5986 | 0.6983 | 0.7353 |
| Accuracy | 0.5872 | 0.5815 | 0.5828 | 0.6154 | 0.6829 | 0.6936 |
| Positive-LR | 1.334 | 1.1053 | 2.2046 | 1.7864 | 1.93 | 1.7128 |
| Negative-LR | 0.7277 | 0.8583 | 0.6275 | 0.6037 | 0.4728 | 0.4638 |
| Diagnose-OR | 1.8332 | 1.2878 | 3.5132 | 2.959 | 4.0821 | 3.6934 |
| N-for-diagnose | 6.6662 | 16.5518 | 3.5148 | 3.7951 | 2.9721 | 3.2677 |
| Positive-pv | 0.802 | 0.7656 | 0.8653 | 0.8404 | 0.8491 | 0.8347 |
| Negative-pv | 0.3115 | 0.2828 | 0.3535 | 0.3598 | 0.4205 | 0.4225 |
| a | 393 | 369 | 302 | 337 | 405 | 414 |
| b | 97 | 113 | 47 | 64 | 72 | 82 |
| c | 263 | 213 | 278 | 226 | 175 | 149 |
| d | 119 | 84 | 152 | 127 | 127 | 109 |
| Abbreviations: AUC, area under curves; ARPG, admission random plasma glucose; GVT, glucose variable tendency; HTN, hypertension; LDL-C, low density lipoprotein cholesterol; PCI, percutaneous coronary intervention; PE, plaque erosion; PR, plaque rupture; ROC, the receiver operating characteristic curve; TIMI, thrombolysis in myocardial infarction. Combination I for: ARPG or GVT, combined with age; gender; HTN history; current smoking; LDL-C. Combination II for: ARPG or GVT, combined with age; gender; HTN history; current smoking; LDL-C; initial TIMI flow; multivessel culprit, and culprit vessel. | | | | | | |

| **Table S3.the correlation between glucose-related variables and plaque morphological characteristics using Spearman correlation** | | | | | | | |
| --- | --- | --- | --- | --- | --- | --- | --- |
| Variables | | HbA1c | ARPG | PFPG | A/C | GVT | DM |
| QCA | Minimum lumen diameter | -0.0446 | -0.0688 | -0.0487 | -0.0375 | 0.0355 | -0.0626 |
|  | Reference vessel diameter | -0.0891 | -0.0711 | -0.0608 | -0.0442 | 0.0279 | -0.0971 |
|  | Degree of stenosis (%) | -0.0227 | 0.0315 | 0.0155 | 0.0252 | 0.0018 | 0.0061 |
|  | Lesion length | -0.0587 | -0.0225 | 0.0058 | -0.0081 | -0.0302 | 0.0147 |
| OCT | Lipid length | 0.0718 | 0.0368 | 0.0455 | -0.0061 | -0.078 | 0.0697 |
|  | Minimal FCT | 0.074 | 0.0512 | 0.0727 | 0.0348 | -0.0218 | 0.0244 |
|  | TCFA | 0.1138^*^ | 0.0801 | 0.0903 | 0.0283 | -0.0402 | 0.0829 |
|  | Mean lipid arc | 0.1243^*^ | 0.1062^*^ | 0.1082^*^ | 0.0589 | -0.0159 | 0.0732 |
|  | Max lipid arc | 0.1219^*^ | 0.0848 | 0.0854 | 0.0219 | -0.0293 | 0.0588 |
|  | Cholesterol crystal | 0.0181 | 0.0403 | 0.0299 | 0.0149 | -0.0752 | 0.0537 |
|  | Microchannel | -0.0237 | -0.0108 | -0.0387 | 0.0323 | -0.0307 | -0.0237 |
|  | Thrombus length | 0.049 | 0.0105 | 0.018 | -0.0163 | -0.0139 | -0.0369 |
|  | Thrombus type | 0.0443 | 0.0134 | 0.0517 | -0.0059 | -0.0173 | 0.0033 |
| Data presented as Spearman correlation coefﬁcient (r). *, P value<0.05. Abbreviation: A/C, admission/chronic glycemic ratio; ARPG, admission random plasma glucose; DM, diabetes mellitus; FCT, thickness of fibrotic cap; GVT, glucose variable tendency; HbA1c, glycosylated hemoglobin; max-, maximal; min-, minimal; OCT, optical coherence tomography; PCI, percutaneous coronary intervention; PFPG, post-PCI fasting plasma glucose; QCA, quantitative coronary angiography; TCFA, thin-cap fibroatheroma. | | | | | | | |
